# An analysis of *exo-*polygalacturonase bioprocess in submerged and solid-state fermentation by *Pleurotus ostreatus* using pomelo peel powder as carbon source

**DOI:** 10.1186/s43141-020-00061-7

**Published:** 2020-09-07

**Authors:** Kausik Majumder, Bubai Paul, Rakhi Sundas

**Affiliations:** 1 Post-Graduate Department of Botany, Darjeeling Government College, Government of West Bengal, Darjeeling, West Bengal 734101 India; 2 Present address: Department of Botany, Bidhannagar College, Government of West Bengal, EB-2, Sector-1, Salt Lake City, Kolkata, West Bengal 700 064 India

**Keywords:** Polygalacturonase, *Pleurotus*, Pomelo peel powder

## Abstract

**Background:**

As there has been an increasing trend in the effective utilization of plant and crop residues for microbial transformation into a desired product, an attempt was made to compare of *exo-*polygalacturonase production using logistic and Luedeking-Piret kinetic model by *Pleurotus ostreatus* in submerged (smf) or solid-state fermentation (ssf) using pomelo peel powder, an agro-forestry residue as carbon substrate.

**Results:**

Cultures grown in submerged fermentation produced a peak of *exo*-polygalacturonase activity as 6160 Ul^-1^ on the 4th day of culture as compared with 2410 Ul^-1^ on the 5th day of fermentation by solid-state fermentation. The enzyme yield coefficient (Y_E/X_) is of higher value in smf vs. ssf system (Y_E/X_ = 1.05 × 10^3^ vs. 0.622 × 10^3^) indicating the more efficient product yield in smf as compared with ssf. The plots derived from*λ* versus*ζ* clearly demonstrate that the secondary product destruction is higher in smf than in ssf.

**Conclusion:**

*P. ostreatus* performs much better in submerged fermentation as compared with solid-state fermentation in respect to *exo*-polygalacturonase production although ssf technique produced a more thermo-stable *exo*-polygalacturonase in crude extract, which is highly desirable in various industrial applications.

## Background

Microbial pectinolytic enzymes of fungal origin have many industrial applications viz food processing, textile, etc. with tremendous potentials [1]. Filamentous fungi, e.g., *Aspergillus niger* are the most frequently used microorganism in the enzyme industry since they produce about 90% of enzyme extra-cellularly. Research reports are available on *exo*-polygalacturonase production by *Aspergillus* species using a wide range of substrates through either ssf or smf. Moreover, comparative assessment between these two techniques, i.e., smf and ssf on the production of polygalacturonase are also reported [2, 3] in which *Aspergillus niger* was used. There are very few works available about the production of polygalacturonase from the edible fungi, such as *Lentinus edodes* [4] or *Pleurotus ostreatus* [5]. These studies are confined either submerged or solid-state fermentation, although no research work has been on record about the comparative assessment on the kinetics of *exo-*polygalacturonase (*exo*-PG, EC 3.2.1.67) production by these two techniques. Moreover, enzymes obtained from the edible fungi can be suitably employed in food processing industries. In recent years, there has been an increasing trend in the effective utilization of crop residues for microbial degradation and transformation into a specific desired product of industrial values and reports are also available on the production of *exo*-polygalacturonase using various agro-industrial residues. Searching for a novel cheap carbon substrate, therefore, is a prerequisite for the effective industrially important enzyme production system. Pomelo (*Citrus maxima*), a member of the family Rutaceae is a rich source of pectin and the yield of pure pectin obtained from this peel is 80.88% and it is rated as high methoxyl pectin (HMP) (DE = 92.75%) with a low viscosity [6]. Both the fermentation systems, i.e., smf and ssf have been applied for the production of enzymes such as esterase, invertase, tannase, β-fructofuranosidase, and *exo-*pectinase, although no comparative kinetic studies have been done to explain the differential behavior of micro-organisms for *exo*-polygalacturonase production in smf and ssf. A number of agro-industrial residues such as tomato pomace, wheat bran, grape pomace, cassava bagasse, etc. are used in solid-state fermentation. Application of a kinetic model, however, has so far been limited, due to the biodegradable nature of these solid supports which prevent correct biomass measurement. The use of an appropriate inert, non-biodegradable support such as polyurethane foam (PUF) can definitely overcome this problem. In the present research, an attempt is made to compare the productivity of *exo-*polygalacturonase of *P. ostreatus* between smf and ssf by applying the logistic and Luedeking-Piret equation for estimation of various growth and production-related coefficients. This study has also focused on the physico-chemical properties of crude *exo-*polygalacturonase, produced by the two kinds of fermentation techniques.

## Methods

### Micro-organism and fermentation system

The mycelial culture of mushroom *Pleurotus ostreatus* was grown at 28 ± 1 °C for 5 days in a medium containing 1% glucose, 1% malt extract, 10% potato extract, and 0.15 % KH_2_PO_4_ after inoculation with small mycelial pieces of the fungus. The enzyme production medium contained (g l^−1^) NH_4_H_2_PO_4_– 24, MgSO_4_.7H_2_O– 0.5, CaCl_2_.2H_2_O– 0.37, H_3_PO_4_– 0.57, FeSO_4_.7H_2_O– 0.25, MnCl_2_– 0.032, NaMoO_4_– 0.032, KH_2_PO_4_– 1.5 [7] in combination with a carbon source at 20 g l^−1^. Mycelial pellets were then transferred aseptically to a 250 ml of polypropylene flask containing two 1-cm diameter glass beads and crushed to form a slurry mixture by shaking for 2 h. For submerged fermentation (smf), 0.4 gl^−1^ inoculums were added to a 50 ml enzyme production medium in a 250-ml Erlenmeyer flask, while for solid-state fermentation (ssf), the same quantity of inoculum was added to polyurethane foam (PUF) cubes of 1 g, absorbed with 50 ml of enzyme production medium in a 250-ml Erlenmeyer flask [8]. Erlenmeyer flask for solid-state fermentation containing PUF with enzyme production media was initially moistened with 75% and incubated at 85 % of the relative humidity of the incubation chamber. Fermentation was carried out for 7 or 10 days at under constant shaking at 180 rpm for smf or on solid matrix-assisted ssf under the static condition at 30 ± 1 °C.

### Carbon substrate

**Fig. 1:**
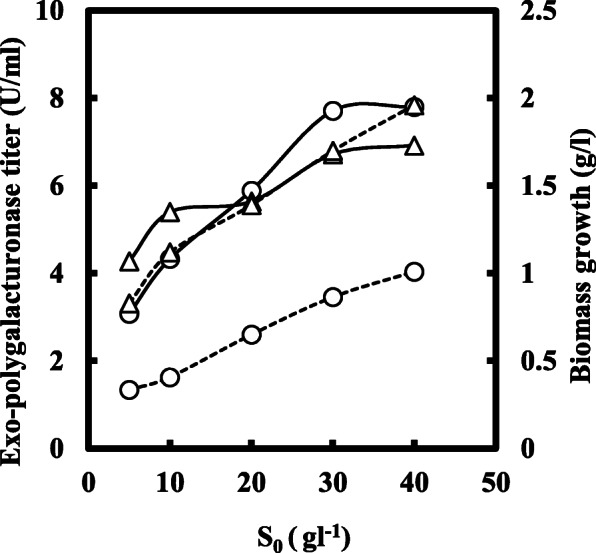
Production trend of *exo*-polygalacturonase titer (○) and biomass (∆) in submerged (−) and solid-state (…) fermentation of *P. ostreatus* for 7 days, with the varying initial level of carbon substrate (pomelo peel powder) in the culture medium. Data presented were the mean value of 3 sets of identical experiments (standard deviation was within ±7%)

The white portion of the peel of pomelo (*Citrus maxima*) was initially washed by water followed by dist. water and oven-dried at 50 °C. The dried mass was grounded into a fine powder and screened through 200 meshes. The sieved mass was dried overnight at 50 °C and used as the carbon source due to its water-soluble nature. The carbon source was used at 20 gl^−1^ in all experiments of smf or ssf except the experiment of Fig. 1, where the varying concentration of carbon substrate was used. All other chemicals used were of analytical grade.

### Preparation of inert supporting matrix

Low-density polyurethane foam (17 kg m^−3^) was used as an inert support and PUF cubes (0.5 cm^−3^) were washed with dist. water, oven-dried at 60 °C [9]. Dried PUF cubes (1 g) were absorbed with liquid medium (50 ml) were then placed in a 250-ml Erlenmeyer flask. The PUF containing culture medium was sterilized by autoclaving under standard conditions [8].

### Enzyme extraction and biomass evaluation

The culture filtrate containing enzymes for submerged fermentation (smf) was obtained by centrifugation of the medium at 10,000 rpm at 4 °C for 10 min. The mycelia were washed with dist. water, collected by filtration using filter paper (Whatman no 1), and dried at 60 °C. For solid-state fermentation (ssf), the PUF cubes with attached mycelium were squeezed to remove the medium containing extracellular enzymes. The culture filtrate was collected by centrifugation under the above-mentioned conditions [8]. After extraction, PUF cubes were thoroughly washed with dist. water to remove any adhered particle with mycelium, immobilized with solid PUF cubes, and dried completely at 60 °C. In both the fermentation systems, biomass was evaluated as the difference of pre-weighed filter paper or pre-weighed PUF cubes [10].

### *Exo*-polygalacturonase activity assay

*Exo-*polygalacturonase (*exo*-PG) activity was assayed by incubating the enzyme for 10 min at 50 °C with 0.5 % (w/v) polygalacturonic acid in 50 mM citrate buffer (pH 6.0). The released reducing groups, expressed as galacturonic acid were quantified by di-nitro salicylic acid reagent [11]. A control was simultaneously prepared taking thermally denatured enzyme. The concentration of the product (D-galacturonic acid) was compared with a D-galacturonic acid standard curve. The enzyme activity was expressed as Uml^−1^ in which, one enzyme unit is defined as the amount of enzyme required for releasing of 1 μmol of galacturonic acid per minute under the assay condition. Total carbohydrate as a substrate in the culture medium was determined by anthrone reagent following standard protocol and the yield coefficient was calculated from the experimental data based on the Monod equation.

#### Theory of bioprocess modeling

In a fermentation system, the microbial growth curve assumes a sigmoid shape and the growth curve can be predicted using Velhurst-Pearl logistic equation [12] as

1 $$ dX/ dt={\mu}_{\mathrm{max}}X\left(1-X/{X}_{\mathrm{max}}\right) $$

Where *μ*_max_ is the maximum specific growth rate (h^−1^) and *X*_max_ is the maximum attainable biomass concentration (gl−^1^)

The integrated form of Eq. (1) with the initial condition *X* = 0 at *t* = 0 which gives as

2 $$ X={X}_0^{e^{\mu \max\;t}}/1-\left({X}_0/{X}_{\mathrm{max}}\right)\left(1-{e}^{\mu\;\max }t\right) $$

The kinetic parameter *μ*_max_ can be determined after rearranging Eq (2) as

3 $$ {\ln}^{X_{\mathrm{max}}}/{X}_0={X}_{\mu }t-\ln \left(\zeta /1-\zeta \right) $$

where *ζ* = *X*/*X*_max,_ i.e., the dimensionless variable of relative growth

If the experimental data is well-fitted in the equation, then a plot of ln(*ζ*/1 − *ζ*) versus time (*t*) give a straight line of the slope, *μ*_max_ and the intercept is − ln(*X*_max_/*X*_0_) [13]

Among the various model reported in the literature to express quantitatively the production rate of a metabolic compound, the classic Luedeking and Piret kinetic model [14] has a wide application in the microbial fermentation system. Kinetics of product formation, i.e., enzyme (E) can be mathematically expressed as

4 $$ dE/ dt={\alpha}^{dX}/ dt+\beta X $$

where *α* and *β* are empirical coefficient that may vary with fermentation conditions. It states that the product formation rate varies linearly with both the instantaneous cell mass concentration (*X*) and growth rate (*dX*/*dt*).

If we consider the product yield coefficient of the enzyme (E) in terms of biomass (*Y*_*E*/*X*_), then the Eq. (4) can be expressed as

5 $$ dE/ dt={Y}_{E/X} dX/ dt+\beta X $$

The coefficient *β* may be negative, positive or zero value, because Luedeking-Piret model is related to growth-associated or non-growth-associated product formation.

The Eq. (5) may be arranged as a function of biomass (where *E* = *E*_0_ at *t* = 0) which gives as

6 $$ {E}_{(t)}={E}_0+{Y}_{E/X}\left(X-{X}_0\right)+{\beta}^{X_{\mathrm{max}}}/{\mu}_{\mathrm{max}}\kern0.5em \ln \left[\frac{X_{\mathrm{max}}-{X}_0}{X_{\mathrm{max}}-X}\right] $$

at a respective time (*t*) of fermentation [15]

Defining *λ* = (*E* − *E*_0_) and *ζ* = *X*/*X*_max,_ the Eq. (6) can be re-arranged in the equitation as

7 $$ \lambda ={Y}_{E/X}{X}_{\mathrm{max}}\left\{\left(\zeta -{\zeta}_0\right)+\sigma \ln \left[\frac{1-{\zeta}_0}{1-\zeta}\right]\right\} $$

where *σ* = *β*/*Y*_*E*/*X*_*μ*_max,_ i.e., the ratio between the rate of secondary formation or breakdown of the product as related to the maximal rate of product formation (*Y*_*E*/*X*_*μ*_max_).

The variable *λ*, which represents the increase of product formation is very important since the plot derived from *λ* versus *ζ* as Eq. (7), the shape of the production curve and the presence of product breakdown can easily be determined and compared between two types of fermentation system [15].

#### pH and temperature stability of crude *exo*-polygalacturonase

In the determination of pH stability, crude *exo*-polygalacturonase was diluted with 0.1 M of different buffer system (pH 3.0–pH 9.0) and incubated at 37 °C for 2 h. The residual enzyme activity was determined following the standard method. The temperature stability of the enzyme was also determined by measuring the residual activity at different intervals after incubating the enzyme at different temperatures (30–70 °C) in 50 mM citrate buffer (pH 6.0).

## Results

### Enzyme production kinetics in submerged vs. solid-state fermentation technique

**Fig. 2:**
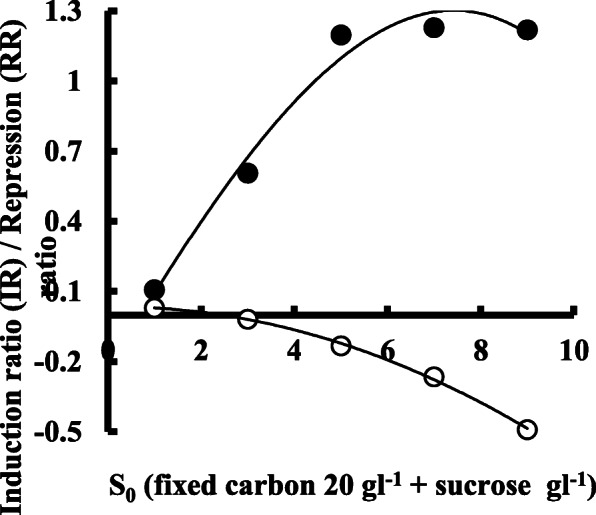
*Exo*-polygalacturonase induction or repression ratio at varying level of sucrose with a fixed concentration of pomelo peel powder in submerged (○) or solid-state fermentation (●) of *P. ostreatus*

**Fig. 3:**
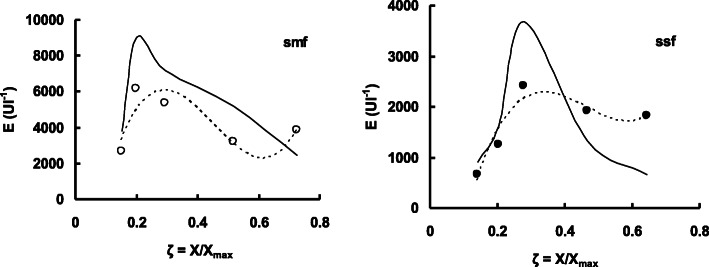
Production trends of exo-polygalacturonase titers, E (Ul−^1^) at a function of various levels of relative biomass production, ζ = X/X_max_ in submerged (○) or solid-state fermentation (●) of *P. ostreatus*. Symbols correspond to the experimental data and solid line (−) correspond to the calculated values by the Luedeking and Piret model together with the logistic equation, broken lines (…) represent the best-fitted line for smf (*y* = 223610x^3^−303547x^2^ + 119806x−8605, *R*^2^ = 0.719) or ssf (*y* = 75729x^3^−105128x^2^ + 45194x−3905, *R*^2^ = 0.898) between the relative biomass and the enzyme titer

**Fig. 4:**
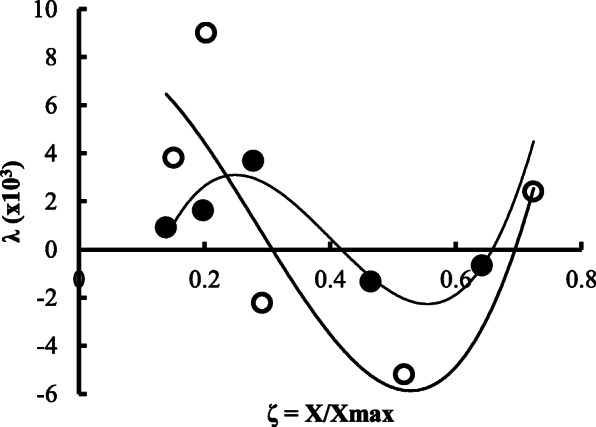
Shape of production curve and presence of product breakdown of *exo*-polygalacturonase at a function of various levels of relative biomass production, ζ = X/X_max_ in submerged (○) or solid-state fermentation (●) of *P. ostreatus*

Enzyme production was affected in a different way by the initial level of the substrate (S_0_) in both types of fermentation systems. In the submerged technique, enzyme titer was about 125% higher as compared with the production in solid-state fermentation (Fig. 1). Moreover, enzyme production shows an increased level of about 80% when substrate concentration was increased by 10 gl^-1^ to 30 gl^-1^ in smf. It is also evident from Fig. 1 that biomass growth was not significantly affected by the type of fermentation performed, although unit production of the enzyme was as 4177 Ug^−1^of biomass and 1870 Ug^−1^in smf and ssf respectively at 20 gl^−1^ of S_0_. However, enzyme titer showed no significant increase above 3% of the initial carbon substrate in both the fermentation employed. Figure 2 shows the effect of the initial sucrose level on the titers of *exo*-polygalacturonase as induction or repression ratio in submerged and solid-state fermentation. It is evident that addition of sucrose (5 gl^−1^) in culture medium containing pomelo peel powder induced an enzyme production as induction ratio of 1.198 in solid-state fermentation, while a repression ratio of −0.133 was observed in culture filtrate enzyme production of submerged fermentation technique at the same initial level of sucrose in the culture medium. If we observe data from Fig. 3, it clearly demonstrates that in smf system, enzyme production reached as 6160 Ul^−1^ on the 4th day of fermentation when the variable of biomass growth as 0.198 while 2410 Ul^−1^ of enzyme activity was observed on the 5th day of culture or *ζ* value of 0.278 in case of solid-state fermentation. However, in both cases, enzyme production was decreased with the progress of fermentation. The bioprocess modeling on the kinetics of product formation helps to compare the experimental production with the predicted theoretical values of enzyme production for both the systems. Now, if the predicted theoretical values of enzyme production were considered, then it is also observed that (Fig. 3) 9040 Ul^−1^ of enzyme activity and 3690 Ul^−1^ of enzyme activity were found on the 4th day of smf and on the 5th day of ssf respectively. Figure 4 of *λ* versus *ζ* as Eq. (7) states that the secondary product destruction is observed maximal in smf as −5.190 × 10^3^ Ul^−1^ on the 7th day as compared with −1.336 × 10^3^ Ul^−1^ in ssf on the 7th day of fermentation. It was also evident from the bioprocess modeling that product destruction was much higher in submerged fermentation.

### Effect of pH and temperature on crude *exo*-polygalacturonase activity and stability

**Fig. 5:**
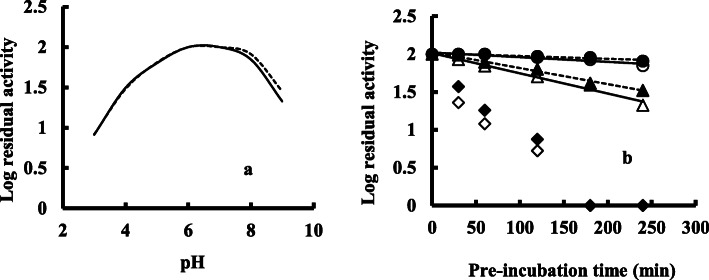
Effect of pH on the stability (**a**) of the crude *exo*-polygalacturonase, produced by *Pleurotus ostreatus* in submerged (−) or solid-state (…) fermentation and the effect of temperature on the stability (**b**) of the crude *exo*-polygalacturonase, produced by *Pleurotus ostreatus* in submerged [(○)- 30 °C, (∆)- 50 °C, (◊)- 70 °C, (−)] or solid-state [(●)- 30 °C, (▲ )- 50 °C, ()- 70 °C, (…)] fermentation. Data presented were the mean values of the 3 sets of experiments (standard deviation of the relative and residual enzyme activity were within ±8% and ±7 % respectively)

Experiments on the pH stability (Fig. 5a) show that *exo*-PG of *Pleurotus ostreatus* was very stable at pH 6.0–7.0 and retained about 60 and 70% of initial activity at pH 5.0 and 8.0 respectively. However, the enzyme lost about 70 and 80% of activity at pH 4.0 and 9.0 respectively. Thermal stability experiment (Fig. 5b) revealed that the enzyme obtained by smf technique retained about 70% after 1 h with an 80% loss of enzyme activity after 4 h at 50 °C. However, enzymes obtained by ssf showed an 80% retention activity and 30% residual activity after 1 and 4 h at 50 °C respectively.

## Discussion

Enzyme production is a challenging field of microbial biotechnology, particularly when the two techniques, i.e., submerged and solid-state fermentation has been well established to produce a wide variety of extracellular enzymes. A number of agro-residues have been exploited for enzyme production using a wide variety of microorganisms in both the fermentation systems, considering its potential for the production of hydrolytic enzymes. Most enzyme manufacturers produce enzymes using submerged fermentation technique, although, solid-state fermentation technique for enzyme production has been significantly increased. Solid-state production of polygalacturonase in *Aspergillus sojae* showed that crushed maize was a good substrate for enzyme production and 48% more polygalacturonase activity was observed in solid-state as compared with submerged fermentation technique [16]. Maldonado and Strasser [2] reported that *Aspergillus niger* produced 6 times higher polygalacturonase in the solid-state system as compared with submerged fermentation using pectin as a carbon source and also require a shorter time for enzyme production. Solid-state fermentation was found to be more suitable (60.20%) for polygalacturonase production by *Aspergillus niger* compared with the submerged fermentation (39.80%) using banana peel as a carbon source [3], while fruit processing wastes including apple pomace, strawberry pomace, etc. are significantly utilized in polygalacturonase production by *Lentinus edodes* in ssf [4]. All of the previously reported literature used solid substrate as a supporting as well as carbon source. However, in the present study, PUF and pomelo peel powder were used as an inert supporting matrix and carbon substrate respectively. The research reports are also available in polygalacturonase production by *P. ostreatus* using tomato pomace [5] and lemon peel waste [17], although those studies had exploited the smf technique only for enzyme production. The experimental results thus showed that pomelo peel powder is a good option for the production of *exo-*polygalacturonase, although, a remarkable difference among two fermentation technique was observed in regard to enzyme production in the present study and the *exo-*PG production is in favor of smf, which is supported by Tellez-Tellez et al. [9], who observed this type of behavior of laccase production in submerged vs. solid-state fermentation. Moreover, enzyme production was significantly increased in PUF based solid-state culture by *Aspergillus niger* as compared with solid-state fermentation on biodegradable support [10].

Sucrose in combination with other carbon sources plays a role in extracellular enzyme production which was also involved with the fermentation techniques. Sucrose improved *exo-*polygalacturonase production in ssf system while a marked inhibition was observed in smf (Fig. 2), supported by Ketipally et al. [18] who observed that addition of sucrose to orange peel media improved the polygalacturonase production in solid-state fermentation of *Aspergillus nomius.* Enzyme induction ratio of tannase [19] and xylanase [20] in the presence of sucrose was also reported to increase in solid-state fermentation. Viniegra-Gonzalez et al. [15] concluded that a marked inhibition of pectinase production was observed in submerged culture of *Aspergillus niger* in the presence of sucrose, although Botella et al. [21] reported that *exo*-PG activities were increased by the addition of extra carbon source in grape pomace-based solid-state fermentation, which also supports the present study of *exo-*polygalacturonase production.

In order to compare the enzyme production kinetics between the two techniques, Logistic and Luedeking-Piret mathematical model was fitted to the observed experimental data considering errors between the observed experimental values and corresponding calculated values. The observed as well as predicted theoretical values of enzyme production (Fig. 3) thus indicated that pomelo peel powder is a good choice as an excellent carbon substrate for *exo*-polygalacturonase production in both systems by *P. ostreatus*. Freixo et al. [5] reported that *P. ostreatus* produced an activity peak of 2181 Ul^−1^ of culture both on the 4th day of fermentation with a specific activity of 42.8 Umg^−1^ of protein in submerged fermentation with tomato pomace as the sole carbon source, which also supports our study. The decrease of enzyme titer (38% lower on the 10th day of culture as compared with highest level) in the advancement of fermentation (Fig. 3) was also supported by Levin and Forchiassin [22], who reported that *Coriolus troggi* secreted polygalacturonase about 2-fold lower on the 15th day of culture. The rate of substrate utilization by fungi generally decreased with the progress of fermentation. The higher value of biomass yield coefficient (Y_X/S_ = 0.255 vs 0.233 smf and ssf respectively) indicates that the substrate was consumed and biomass production was faster and more efficiently in smf as compared with ssf technique. The enzyme yield coefficient on unit biomass (Y_E/X_) is of higher value in smf system (Y_E/X_ = 1.05 × 10^3^ vs. 0.622 × 10^3^) which indicates the more efficient product yield per unit biomass in smf as compared with ssf technique. Harvesting time, therefore, is a crucial factor in fermentation since extra-cellular enzyme was broken down in proteolysis [19] while lowering of carbon substrate favored the protease production at the end of the culture period. In the modeling of the fermentation technique for enzyme production (Fig. 3), the theoretical titer of enzyme production was calculated by Velhurst-Pearl and logistic equation, with high correlation coefficient (*R*^2^ > 0.98) as a function of the relative degree of advancement of the fermentation (ζ = X/X_max_). The plots derived from *λ* versus *ζ* as Eq. (7) clearly demonstrate that the secondary product destruction is higher in smf as compared with ssf, although the maximum value of enzyme yield present in smf system (Fig. 4). This result has practical significance because *λ* the variable which represents the increase of product formation is a major parameter for industrial production of enzymes and it is also observed that in both cases, enzyme decay is present (Fig. 4), although the magnitude of decay was higher in submerged condition [15]. The breakdown of *exo*-polygalacturonase by contaminant protease in the fermentation period may be a possible explanation for the enzyme decay. The present research showed higher growth and greater production of *exo*-polygalacturonase in smf as compared with ssf supported by Tellez-Tellez et al. [9] who reported that lacasse production by *P.ostreatus* was higher in smf culture than in PUF-based ssf culture.

The critical physiological and kinetic studies are therefore the basis for efficient process development control strategies as well as downstream processing. The success or failure of a bioprocess depends upon a number of factors. The submerged fermentation works as a homogenous system require large energy expenditure to supply oxygen at fast rate to cope with high oxygen demand during enzyme production, although solid-state fermentation has added advantage of being a static process requiring minimum energy expenditure, although enzyme production by ssf for large scale operation, rotating drum bioreactor was used [23]. The breakdown of the enzyme at the later phase of fermentation (Fig. 4) is probably responsible for lowering enzyme level in both the systems, although the higher magnitude of enzyme breakdown was evident in smf as compared with ssf may be due to substrate utilization, presence of other metabolites or low pH value in the culture filtrate [24]. Viniegra-Gonzalez et al. [15] reported that the production of protease was a secondary and undesirable outcome of pectinase production and a lower protease level was observed in *Aspergillus niger* mediated solid-state culture of pectinase production as compared with smf. However, future research with microscopic image analysis of fungal growth for both systems, contaminant protease level in culture medium and evaluation of the effect of oxygen on the enzyme production would be necessary to clarify this differential behavior of *exo*-polygalacturonase production by *P. ostreatus* in submerged and solid-state fermentation.

From the pH and thermal stability experiments (Fig. 5), it was observed that the inactivation process of *exo*-PG is faster at acidic pH as compared with alkaline pH. Crude *exo*-polygalacturonase, however, exhibited a quite broad range of pH stability, supported by Tari et al. [25], in which crude *exo*-PG of *Aspergillus sojae* also stable at pH 5.0–7.0. The PG produced by *Lentinus edodes* was fairly stable between pH value of 3.0 and 6.5 [4]. The results indicate that *exo*-PG of *P. ostreatus* prefers towards slightly alkaline pH, which is highly appreciable for industrial purposes. Thermo-stability of pectin degrading enzymes is an important property, particularly applicable in fruit processing industries. The present findings, therefore, revealed that exo-PG produced by *P. ostreatus* through ssf technique is more thermally stable as compared with smf technique, which is also supported by Acuna-Arguelles et al. [26], in which pectinases obtained by *Aspergillus oryzae* cultivation in solid-state fermentation were more resistant to pH and temperature changes compared to those obtained by submerged fermentation. Rashad et al. [17] reported that purified PG produced by *P. ostreatus* retained about 93% activity at 40 °C and exhibited 100% of its initial activity at 30 °C. The thermo-stability of the purified polygalacturonase of *P. ostreatus* was reported to be stable at pH 6.0 as compared with 7.0 at 50 °C [5]. However, the crude *exo*-polygalacturonase of *P. ostreatus* was more stable thermally than purified one, may be due to protein-protein interaction in the culture filtrate, secreted by the fungi [27].

## Conclusion

The choice of a substrate is of great importance for the successful production of *exo*-polygalacturonase. Since the cost of the substrate plays a crucial role in the economics of enzyme production, cheap agro-residues are assessed and pomelo peel powder is an effective option for the *exo*-polygalacturonase production as sole carbon source. The findings showed that *P. ostreatus* performs much better in submerged fermentation as compared with solid-state fermentation in respect to *exo*-polygalacturonase production. However, the ssf technique although produced a lower yield of enzyme as compared with smf but it could be able to produce a more thermo-stable crude *exo*-polygalacturonase, which is highly desirable in various industrial applications.

### Nomenclature

X  biomass concentration at time *t* [g l^−1^]

X_0_  biomass concentration at time *t* = 0 [g l^−1^]

S_0_ carbon substrate concentration at time *t* = 0 [g l^−1^]

μ_max_ maximum specific growth rate [h^−1^]

X_max_ maximal biomass concentration at time *t*→∞ [g l^−1^]

Y_X/S_  yield coefficient for biomass production on substrate consumed [Xg^−1^]

Y_E/X_ yield coefficient for enzyme production on unit mycelial biomass [Ug^−1^]

*β *secondary coefficient of enzyme production or degradation

*λ  *represent the increase of product formation

*σ *ratio between the rate of secondary production or denaturation of enzyme related to the maximal rate of enzyme production

*ζ* dimensionless variable of growth

## Data Availability

Data is available with this article.

## Abbreviations

smf: Submerged fermentation
ssf: Solid-state fermentation
*exo*-PG: *Exo-*polygalacturonase
HMP: High-methoxyl pectin (HMP)
DE: Degree of esterification
PUF: Polyurethane foam
